# Bi-valvular endocarditis occurring 2 months after COVID-19 infection

**DOI:** 10.11604/pamj.2021.39.37.29540

**Published:** 2021-05-13

**Authors:** Salma Kraiem, Hassen Ibn Hadj Amor

**Affiliations:** 1 Department of Cardiology, Taher Sfar Hospital, Mahdia, Tunisia

**Keywords:** Bivalvular endocarditis, vegetation, COVID-19

## Image in medicine

A 60-year-old diabetic man was admitted in cardiology department for dyspnea and fever evolving over 3 weeks with urinary symptoms. In his past history there was a COVID-19 infection that required hospitalization with oxygen therapy for a week and he reported repetitive urinary tract infections. The physical exam showed an axillary temperature at 39.5°c crackling sounds on the lung and no right ventricular failure signs. Laboratory tests showed biologic inflammatory markers elevation; cytobacteriological urine test isolated: enterococcus foecalis and several hemocultures isolated the same named bacteria. Transthoracic echocardiography showed a preserved left ventricular function, a vegetation at the expense of the anterior leaflet of mitral valve (7*4mm); mild mitral valve regurgitation and a huge vegetation at the expense of tricuspid valve (15*20mm) with important tricuspid regurgitation and pulmonary arterial hypertension. The transesophageal echocardiography showed the same vegetation at the expense of the mitral and tricuspid valve and no abnormalities in the other valves. After an initial antibiotic therapy, the patient was referred for surgery in front of worsening mitral insufficiency which has become grade 4.

**Figure 1:**
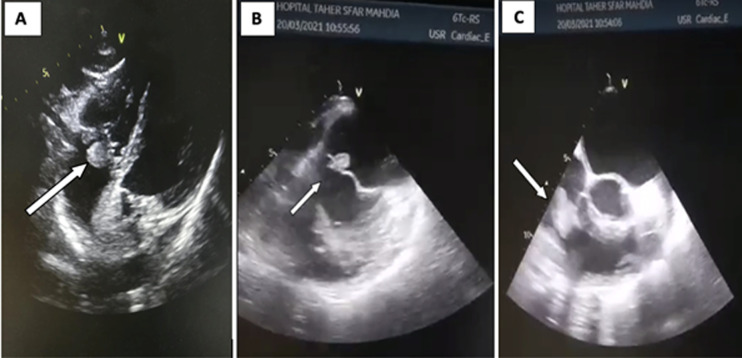
A) huge vegetation at the expense of the tricuspid valve in the subcostal window (15*20mm); B) vegetation at the expense of the mitral valve in transesophageal echocardiography (7*4mm); C) vegetation at the expense of the Tricuspid valve (15*20mm) in the transesophageal echocardiography (TEE)

